# BGFD: an integrated multi-omics database of barley gene families

**DOI:** 10.1186/s12870-022-03846-9

**Published:** 2022-09-21

**Authors:** Tingting Li, Jianxin Bian, Minqiang Tang, Hongbin Shangguan, Yan Zeng, Ruihan Luo, Huifan Sun, Qinglin Ke, Xiaojun Nie, Yihan Li, Licao Cui

**Affiliations:** 1 College of Bioscience and Engineering, Jiangxi Agricultural University, Nanchang, 330045 Jiangxi China; 2 Peking University Institute of Advanced Agricultural Sciences, Weifang, 261325 Shandong China; 3 College of Forestry, Hainan University, Haikou, 570228 Hainan China; 4 State Key Laboratory of Crop Stress Biology in Arid Areas and College of Agronomy, Northwest A&F University, Yangling, 712100 Shaanxi China

**Keywords:** Barley, Gene family, Multi-omics, Database

## Abstract

**Background:**

A gene family comprises a group of genes with similar functional domains that play various roles in plant growth, development, and responses to environmental stimuli. Barley (*Hordeum vulgare* L.) is the fourth most cultivated cereal crop worldwide, and it is an important model species for genetic studies. Systematic identification and annotation of gene families are key for studies of molecular function and evolutionary history.

**Results:**

We constructed a multi-omics database containing 5593 genes of 77 gene families called the Barley Gene Family Database (BGFD: http://barleygfdb.com). BGFD is a free, user-friendly, and web-accessible platform that provides data on barley family genes. BGFD provides intuitive visual displays to facilitate studies of the physicochemical properties, gene structure, phylogenetic relationships, and motif organization of genes. Massive multi-omics datasets have been acquired and processed to generate an atlas of expression pattern profiles and genetic variation in BGFD. The platform offers several practical toolkits to conduct searches, browse, and employ BLAST functions, and the data are downloadable.

**Conclusions:**

BGFD will aid research on the domestication and adaptive evolution of barley; it will also facilitate the screening of candidate genes and exploration of important agronomic traits in barley.

**Supplementary Information:**

The online version contains supplementary material available at 10.1186/s12870-022-03846-9.

## Background

A gene family is made up of homologous genes having a common ancestor and possessing two or more copies that originate from gene duplication [1–3]. Members of the same gene family sometimes can be closely placed to form a cluster of genes. However, most of the time they are distributed in different locations on the same chromosome or scattered across different chromosomes [4]. Gene duplication and loss are primary factors during the dynamic evolution of gene families [5]. Duplications arise mostly through two major processes, small-scale duplications (SSD), such as segmental, tandem, and transposon-mediated, and whole-genome multiplications (WGM) [6]. Initially, the evolutionary outcome of gene duplication is to accelerate excessive redundancy. As the duplicated genes evolve, some accumulate deleterious mutations and are lost, whereas others gain new functions and are permanently preserved, eventually reducing or eliminating redundancy [7].

Genes of the same family have similar structure and function, encoding functionally related protein products with conservative domains [8]. Evidence suggests that gene families are the master regulators for diverse biological processes [9]. Some well-documented examples are transcription factor (TF) gene families, such as heat shock transcription factor (HSF), APETALA2/ethylene-responsive factor (AP2/ERF), NAM/ATAF/CUC (NAC), and basic helix-loop-helix (bHLH). These TF gene families are now known as crucial regulators of various stress responses, e.g., their response to hormones improves plant viability under environmental adversities [10, 11]. In addition to participating in specific stress responses, TF gene families are implicated in stress tolerance, playing a critical role in interconnected stress regulatory networks [12]. Additionally, many TFs are involved in plant growth and developmental processes mediated by plant hormones, such as abscisic acid (ABA), gibberellin (GA), and brassinosteroid (BR) [13, 14]. The involvement of TFs in hormone signaling pathways increases the complexity of the multifaceted regulatory networks [15].

From the origin of agriculture to the present, barley (*Hordeum vulgare* L.) has been the most important temperate crop, ranking fourth among cereals in terms of both farming acreage and tonnage harvested [16]. Approximately 75% of barley global production is used as an ingredient in animal feed, 20% is utilized for the preparation for alcoholic and non-alcoholic beverages, and the remaining 5% is used for a variety of other foodstuffs [17]. Barley is well-adapted to a wide range of harsh environmental conditions, including high salinity, low temperature, and intense ultraviolet exposure in high-altitude areas [18]. Compared to its close relative wheat, barley is stress-tolerant. Consequently, it is a stable source of food for humans in poorer countries, sustainable in marginal and variable environments [19].

The assembly of barley genome has long been lagged due to its high content of transposon elements and large genome size. Thanks to high-throughput sequencing technologies (e.g., chromosome conformation capture (Hi-C), 10X genomics and Bionano Genomics optical map) and advanced algorithms (e.g., TRITEX pipeline), the barley Morex assembly was first released in 2012 [19] and its subsequent revisions have experienced many rounds of improvement (Morex V1 and V2) [20, 21]. It should be of note that the most updated Morex V3 reference genome was generated by PacBio High-Fidelity (HiFi) sequencing, which displayed excellent performance and near-complete coverage in the repeat-rich intergenic regions. Since the same RNA-seq datasets were employed for gene annotation, the gene models of Morex V3 showed almost completed alignments (≥99% identity and ≥95% alignment coverage) with the Morex V2 assembly [22]. Recently, the publication of the first-generation barley pan-genome has also greatly expanded the amount of natural and induced sequence variation available to genetic and breeding studies [23].

With the advent of multi-omics data, more and more gene families have been identified and analyzed in barley. For instance, members of the mTERF gene family are implicated in signaling pathways in response to abiotic stresses [24]. The HAK/KUP/KT potassium transporter gene family is induced by salt, drought, and potassium (K) deficiency stresses [25]. The role of the bZIP TF family as related to starch synthesis has been reported [26]. Many other gene families have been well-documented in barley, such as xyloglucan endotransglucosylase/hydrolases (*XTH*s) [27], non-specific lipid transfer proteins (*nsLTP*s) [28], SQUAMOSA*-*promoter binding like (*SPL*) [29], and *GRAS* (named after the first three identified proteins within this family, GAI, RGA, and SCR) [30] gene families. However, there is no integrated database with large-scale multi-omics data for barley gene families.

To facilitate research on the rapidly growing amount of data, we built the Barley Gene Families Database (BGFD) (http://barleygfdb.com), which contains data on genes from 77 gene families including 37 TF families. BGFD provides basic information on barley gene families, such as their physicochemical properties, chromosomal locations, exon-intron structures, conserved domains, and phylogenetic relationships. Large-scale multi-omics datasets, including 13 transcriptome experiments spanning 413 separate samples, 220 exome-captured sequencing accessions, and 22 newly released reference genomes, facilitate the acquisition of tissue-specific, stage-specific, and stress-induced expression profiles, as well as genomic variation landscapes. The database has an organized and user-friendly web interface. Users can query BGFD to display and search the detailed annotations using gene information, such as gene family names, gene IDs, and genomic regions. This database provides comprehensive information on barley genes and is a useful exploratory tool for functional genomics research and the molecular breeding of barley.

### Construction and content

#### Data resources and identification of gene families

The genomic information of barley reference assembly (Morex V2) was retrieved from the IPK database (https://doi.org/10.5447/ipk/2019/8). Genes are always clustered into families based on their conserved domains [31]. The Hidden Markov Model (HMM) profiles of the 77 gene families were obtained from the Pfam database. For each gene family, the HMM profile was used as a query to search against the barley proteins using HMMER v.3.1 with an *e*-value of 0.001. The putative proteins were further validated using the InterPro (http://www.ebi.ac.uk/interpro/), the National Center for Biotechnology Information–Conserved Domain Database (NCBI-CDD) (http://www.ncbi.nlm.nih.gfov/Structure/cdd/cdd.shtml) and the PFAM (http://pfam.xfam.org) databases. Candidates confirmed by at least one database were retained.

#### Characterization of basic information

The nucleotide sequences, protein-coding sequences, protein sequences, chromosome location, strand, and sequence length were obtained based on the gene transfer format (GTF) file. The physicochemical characteristics, including molecular weight (MW), theoretical point (pI), instability index (II), and grand average of hydropathicity (GRAVY) were calculated using the online tool ExPASy (http://web.expasy.org/protparam/).

#### Phylogenetic relationship, gene structure, and conserved motif analysis

A multiple sequence alignment of full-length proteins was carried out using ClustalW v2.1. A neighbor-joining (NJ) tree was generated using MEGA X with 1000 bootstrap replicates. An online Multiple Expectation Maximization for Motif Elicitation (MEME) was used to detect conserved motif patterns with a maximum number of motifs set at 8 and an optimal motif width range from 6 to 50 amino acids. The intron and exon annotations were obtained from the GTF file and the gene structure was displayed using the Gene Structure Display Server (GSDS) (http://gsds.cbi.pku.edu.cn/). The 1.5 kb sequence, upstream, gene coding regions were extracted and submitted to the online PlantCARE database to detect the *cis-*elements within promoters.

#### Identification of orthologous genes

The protein sequences of rice and *Arabidopsis* were downloaded from the Ensembl Plants database (https://oct2017-plants.ensembl.org/index.html). Orthologous relationships between barley and rice, and between barley and *Arabidopsis* were generated using the program Inparanoid v8.0. The synonymous substitution rate (Ks), non-synonymous substitution rate (Ka), and Ka/Ks ratio were estimated for orthologous gene pairs using codeml of PAML v4.3. The orthologous relationships were plotted using Circos v0.67. Divergence time was inferred using the formula T = Ks/2λ, where T is the time of duplication, Ks indicates the synonymous substitutions per site, and λ is the mutation rate of the divergence of plant nuclear genes (λ = 6.5 × 10^-9^).

#### Expression patterns of barley gene families

A total of 13 publicly available RNA-seq experiments composed of 413 samples with replicates were obtained from the NCBI sequence reading archive (SRA) database. Detailed information for each experiment (accession number, project description, and relevant publication) is given in Supplementary Table 1. Low-quality reads were removed using Trimmomatic v0.39 (https://github.com/usadellab/Trimmomatic). Clean reads were mapped to the barley reference genome using HISAT v2.1.0. Aligned reads were sorted using SAMtools v1.3.1. The fragments per kilobase per million reads (FPKM) of each gene were calculated according to the reference annotation file. The expression level was visualized by the pheatmap package of R.

#### Nucleotide variation identification

The whole-exome sequencing datasets of 220 barley accessions collected worldwide were downloaded from the NCBI SRA database under the BioProject accession number: PRJEB8044 (Exome Capture to Study Genomic Diversity, Adaptation, and Selection in Barley) [32]. Read quality was evaluated and low-quality reads were filtered using Trimmomatic v0.39. The high-quality reads were aligned to the reference genome using BWA-MEM v0.7.13r1126. Picard v1.119 tools were used to clean, sort, and mark PCR duplicates of binary alignment map (BAM) files. Variant calling of BAM files was performed using the Haplotype Caller tool embedded in GATK v3.5-0-g36282e4. Single nuclear polymorphisms (SNPs) with minor allele frequency (MAF) <0.05 or >0.95, or missing rates >0.90 were removed. Only biallelic alleles were retained. Functional annotation and impact on coding regions of variation were determined using SnpEff v4.3. The upstream and downstream region of the gene was set to 3kb.

#### Clustering of ortholog groups (OGs) and syntenic relationships of genes within the barley pan-genome

Publicly available barley genomes were downloaded from the following websites: http://viewer.shigen.info/barley/index.php (wild barley accession “OUH602”) [33], http://viewer.shigen.info/harunanijo/index.php (Japanese elite malting barley cultivar 'Haruna Nijo') [34], and https://barley-pangenome.ipk-gatersleben.de (barley pan-genome project) [23]. This same approach was used to identify gene family members in the barley pan-genome. To make comparisons among family gene repertoires, OGs were identified using OrthoFinder v2.4.0 with default settings, except that the ‘msa’ option was used. The OGs identified were further divided into three parts: core OGs, which represent the set of OGs common to all barley accessions; shell OGs containing accession-specific OGs common to a subset of accessions; and cloud OGs, which are specific to unique barley accessions. Furthermore, the synteny blocks between pairwise genomes were identified between pairwise barley genomes using JCVI-syn2.0 software, which is the Python version of MCSCAN. The shared and specific gene family members between genomes were detected using an in-house script written in Python.

#### Server, operating system, and website construction

The webserver was hosted on a lightweight application server of Tencent cloud (https://cloud.tencent.com/), freely accessible for non-commercial use via its website. Linux system CentOS v7.6.1810 (http://www.centos.org) was installed on the server. The front end of the webpage is implemented in HTML (https://www.w3.org/html/) CSS (http://www.w3.org) and JavaScript (https://www.javascript.com/), and PHP (https://www.php.net/) supported the server-side back-end. Multi-omics data were processed and stored in the MySQL v5.6.50 database server following the MySQL operations manual. JBrowse was installed to provide a user-friendly interface capable of accessing genome information of interest. ViroBLAST constructed a standalone database so online BLAST searches could be performed. Some local scripts were rewritten to provide additional search services.

### Utility and discussion

#### Comprehensive identification and characterization of gene families in barley

**Fig. 1:**
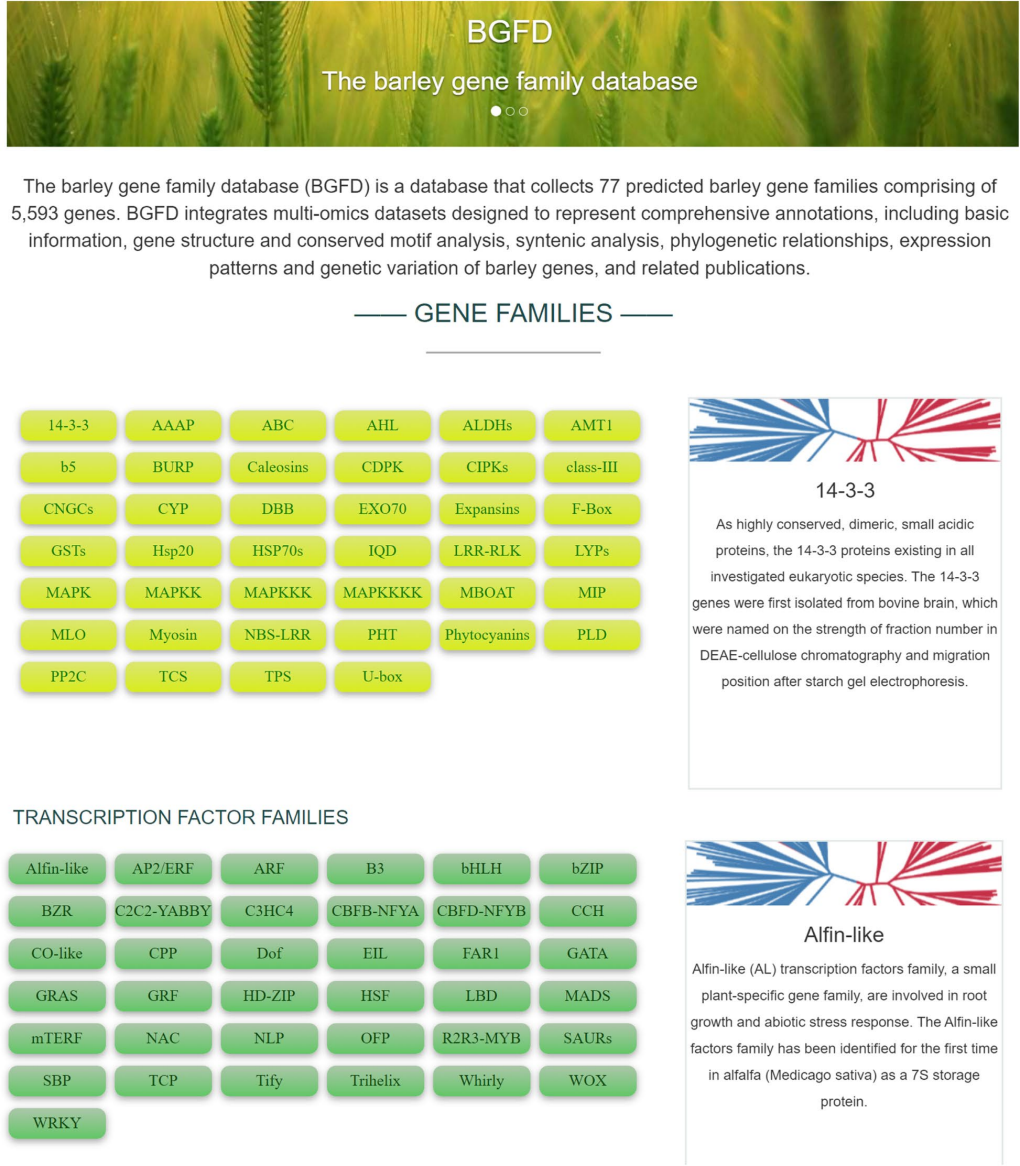
The home page of BGFD

**Fig. 2:**
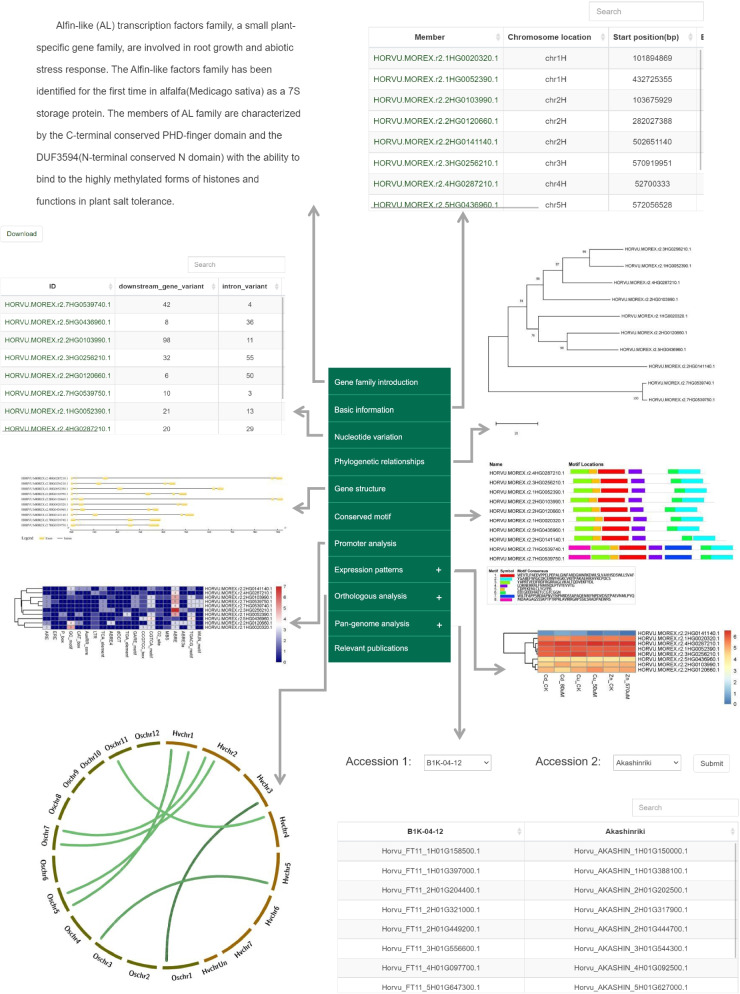
The gene family page of BGFD (e.g., Alfin-like)

BGFD is a database of barley gene families; it provides comprehensive information at both the gene and family levels (Figs. 1 and 2). HMM search and online database validation were used to generate a comprehensive list of gene families. A total of 77 gene families composed of 5593 members were identified. LRR-RLK possessed the largest number of gene family members (502), whereas Whirly had the smallest number (2). The home page for each gene family provides links (on the left navigation menu) to its interfaces along with a brief introduction. Other information, such as gene IDs, chromosomal location, strand, and protein length, is provided for each gene. Each member has a separate display window for other types of information, such as information on physicochemical properties, GO annotations, and alternative gene IDs in the Morex V1 and V3 assemblies. BGFD also provides relevant publications for access to more detailed information. The full text of the related articles can be accessed by clicking on the hyperlinks. BGFD also features a scrolling functionality to facilitate data retrieval.

#### Phylogenic relationships, structure, and conserved motif organization of barley gene families

To further elucidate the evolutionary relationships of specific gene families, multiple sequence alignment was carried out using full-length protein sequences. An unrooted phylogenetic tree was constructed using the neighbor-joining method with 1000 bootstrap replicates. The clustering profile and member assignment can be determined visually. Users can download the multiple alignment outputs in FASTA file format and re-construct the phylogenetic tree based on their personal preferences.

Characterization of the intron-exon structure of genes not only facilitates our understanding of functional diversification within gene families but also provides additional characters that can be used in phylogenetic analyses. Intuitive diagrams of gene structure are provided in BGFD. The black lines and yellow boxes display introns and exons, respectively. The scale bar represents the length at the bottom of the picture. Eight conserved motifs were identified through MEME online tools. The distribution of conserved motifs represents the core regions associated with the biological functions of genes. The colored boxes represent different conserved motifs. Consensus sequences of conserved motifs are shown at the bottom of the webpage. The location of each motif is estimated using the scale at the bottom.

#### Ortholog analysis between Arabidopsis and rice of barley gene families

To provide preliminary information that would aid the cross-referencing and classification of genes from different species, *Arabidopsis* and rice were used to identify orthologs in barley. The number of shared orthologous genes was 43.84% in *Arabidopsis* and 56.16% in rice. The distribution of orthologous gene pairs was consistent with their genetic relationships. Ka/Ks ratios were calculated to characterize the mechanisms underlying the evolution of these gene families. Generally, Ka/Ks < 1, Ka/Ks = 1, and Ka/Ks >1 indicate negative (purifying), natural, and positive selections, respectively. Ka/Ks ratios between barley and rice ranged from 0.001 to 0.7592 with an average of 0.1748; by comparison, the average Ka/Ks ratio was 0.0409 (0–0.4000) between barley and *Arabidopsis*. These orthologous gene pairs can facilitate evolutionary and functional analysis of barley genes.

#### Temporal-spatial and stress-induced expression profiles

Analysis of stage-specific, tissue-specific, and stress-induced expression patterns will serve as a valuable resource for the potential functions of genes in plant species. Expression profiles were quantified using 13 RNA-seq experiments spanning 413 samples from various genotypes, tissues/stages, and abiotic and biotic stress conditions. Expression levels of barley family genes were evaluated by FPKM. The data were presented in a freely available single interface that provided numerical and visual options to profile barley family genes across all the tested RNA-seq datasets. Users can make comparisons between RNA-seq samples, including the expression patterns of genes of interest in different tissues and stages and under different stress conditions. For example, after searching *HORVU.MOREX.r2.2HG0149900* in the caleosin gene family, a tissue-specific pattern of higher expression in the developing grain (15 days after pollination) was observed; however, its expression was low in other tissues/stages. The expression of *HORVU.MOREX.r2.5HG0442310* in the LRR-RLK gene family was up-regulated ~134.64-fold compared with the control under cold treatment. These findings indicate that these genes would make candidate targets for the functional cloning and molecular breeding of barley.

*Cis*-elements play essential roles in the transcriptional regulation of genes throughout the life cycle of plants. To get a preliminary insight into the regulatory mechanism and biological functions of barley family genes, the *cis*-acting regulatory elements within the promoters were integrated into BGFD. A total of 56 kinds of functional *cis*-elements were identified and classified into five categories: hormone-responsive elements, light-responsive elements, organogenesis-related elements, stress-related elements, and structure and composition elements. Twenty functionally important *cis-*elements were displayed using a heatmap. Determining the variety and quantity of regulatory elements could provide insight into the regulatory mechanisms of genes involved in hormone signal transduction, plant growth and development, and responses to abiotic and biotic stress. Data on promoter sequences, categories, and abundances of the *cis*-elements can be downloaded by clicking the “Download” button.

#### Variation analysis of barley gene families

SNPs are the most common type of genomic variation in living organisms [35]. A total of 270,632 high-confidence SNPs were identified from the exome sequencing data of 220 diverse barley germplasms representing 85 wild barley accessions and 135 barley landraces worldwide. The SNPs located in the gene-associated regions, including the upstream, exon, intron, and downstream regions, were retained. The interface summarizes information for the nucleotide variants of each gene. These nucleotide variants can be retrieved in a variant call format (VCF) file. The genetic variants are valuable for molecular marker-assisted selection, genome-wide association studies of important agronomic traits, and research into the domestication and adaptive evolution of barley. For example, *HORVU.MOREX.r2.4HG0282710* (CYP), *HORVU.MOREX.r2.7HG0614640* (NBS-LRR), and *HORVU.MOREX.r2.3HG0252360* (WRKY) were highly divergent between barley landraces and wild barley accessions based on nucleotide variants, and these genes experienced severe genetic bottlenecks during domestication, suggesting that they might be domestication-related candidate genes.

It is now widely agreed that one or a few reference genomes are insufficient for capturing the full range of genetic diversity of a species [36]. The additional barley genomes [33, 34] and pan-genome [23] recently published reveal a high degree of structural variation, including inversions, translocations, copy number variation (CNV), and presence/absence variation (PAV), which facilitates exploration of the alleles of agronomically significant genes. This approach has been used to identify 263,267 gene family members within the barley pan-genome. OG analysis revealed 4099 core, 7405 shell, and 33 cloud OGs within the barley pan-genome. The shell and cloud OGs might be involved in additional biochemical pathways and biological functions, and some of these might be candidate genes that could be explored in future functional investigations, as well as used to genetically improve barley. Large-scale synteny blocks with large numbers of genes were identified between pairwise genomes. The average number of syntenic genes was 5273, which accounts for 94.27% of the reference genome (Morex V2), whereas no syntenic relationships were detected for the rest of the genes (5.73%). These data provide preliminary insights into the structural variation of barley family genes. Users also have the option to determine syntenic relationships by applying different query genomes to the reference genomes.

#### Database implementation and practical tools

**Fig. 3:**
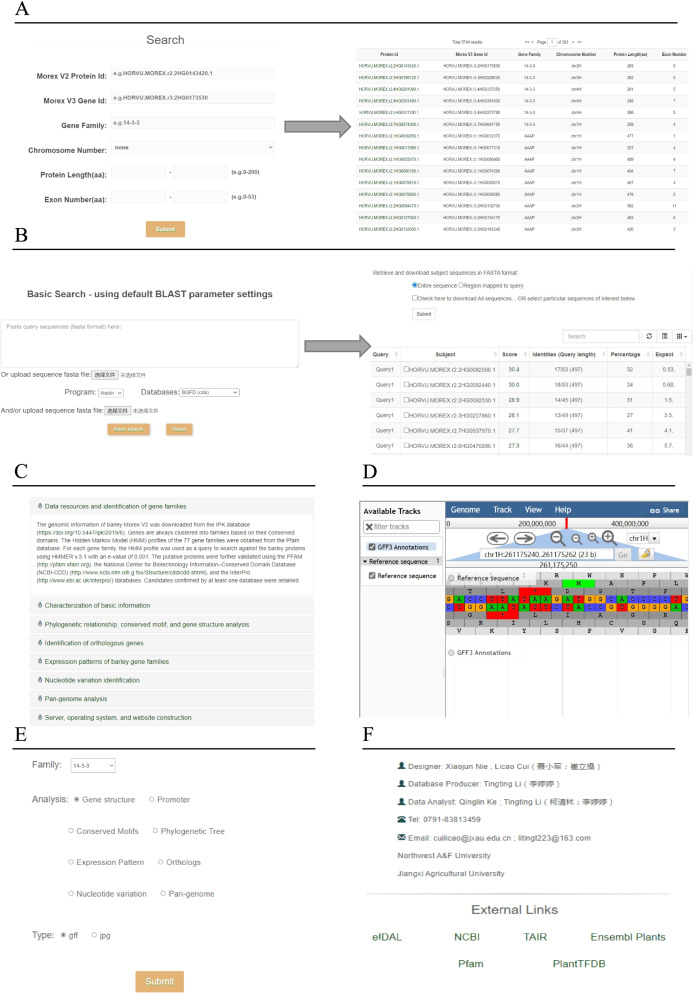
An illustration of the BGFD system. **A** The search functions. **B** Sequence Blast tools. **C** An introduction of BGFD. **D** JBrowse framework. **E** The download pages. **F** The contact information

The BGFD is also implemented with family analysis-related online tools. Our platform contains seven main sections, including the homepage, search tool, website introduction, BLAST service, JBrowse framework, download, and contact information (Fig. 3).

##### Home

The home page consists of three major parts. At the top of the website, we provide a brief introduction of the BGFD, followed by a list of 77 gene families including 37 TF gene families. As the mouse hovers over a specific gene family, a brief introduction of the gene family comes up in the frame to the right.

##### Search

The search function was embedded in BGFD to support various retrieval requirements. For querying gene families or genes, users can search the BGFD by submitting the entire name of the gene family or the gene ID, and even a truncated version can be accepted. In addition, an advanced search model is provided in BGFD. Users can access an interface with a list of eligible genes using search criteria such as chromosome number, protein length, or exon number. The detailed annotation of gene results can be browsed by clicking on the super link of a gene ID.

##### Introduction

The “Introduction” page provides a drop-down menu from which users can browse the “Materials and Methods” used in BGFD. Data resources and official websites of the software can be visited by clicking on the links. The analysis workflow with detailed parameters is also shown on this page.

##### JBrowse

Being universal and customizable, the JBrowse framework was integrated into BGFD to interpret and visualize the genomic features. JBrowse is highly portable and can be configured with data tracks that include Gene, mRNA, CDS (Coding Sequence), and 6-frame translation. With the help of this tool, users can acquire the genomic loci quickly and accurately, allowing breeders to identify candidate genes that are associated with traits of interest.

##### BLAST

The “BLAST” tool allows users to determine all information related to the fragment sequences. For example, if a cDNA fragment is characterized in barley cDNA libraries, the BLAST search tools could be used. Users can submit the query nucleotide or amino acid sequences into the input box in FASTA format or directly upload text files. BGFD supports five basic BLAST algorithms (BLASTN, BLASTP, BLASTX, TBLATN, and TBLASTX). BLAST tools allow users to perform sequence similarity searches against the barley gene family genes. For advance searches, users can set parameters such as expected thresholds, max target sequences, and output format.

##### Download

The entire data resources in BGFD are available for downloading and reanalysis by end-users.

##### About

The “About” page displays some genetic external links that users can access quickly. This site also offers contact and other researcher information. Feedbacks from researchers are welcome and will inform future updates and developments of BGFD.

#### The advantages and features of BGFD

The advances in sequencing technology and bioinformatics play key roles in deciphering complex genomes. New plant genome assemblies, especially for cereal crops, are being released. Comprehensive databases are desired for collecting, storing, and maintaining genomics data for further study of underlying biological functions and molecular mechanisms. The sequencing of the first *Arabidopsis thaliana* genome ushered in a new era for the identification of gene families at the whole genome scale [37]. Several *Arabidopsis* gene family databases are available over the Internet. Approximately 70 families of TFs have been categorized in these public databases, including DATF [38], RARTF [39], and ARGIS [40]. Additionally, other competing databases, such as PlnTFDB [41], PlantTFDB v4.0 [42], GFDP [4], and MGFD [43], collect gene family data of *Arabidopsis* and other plant species. Once enormously helpful and informative, these underrepresented databases now lag behind the steadily updated genomes and multi-omics data. These databases focus on the identification and primary characterization of gene family members, while more useful information for users, such as expression patterns and variation atlas, are not included. To make better use of the multi-omics information for crop research and breeding, it is essential to systematically use multi-omics data from different sources or integrate multi-omics data generated from the same panel.

BGFD has specific advantages and features:

1. BGFD is the first attempt to identify and characterize barley gene families, an effort vitally important to the study of gene biological function and evolutionary history. The BGFD database contains 77 gene families consisting of 5593 genes making it the most comprehensive database for barley gene family research.
2. BGFD integrates several generic database sources, including IPK, Pfam, Expasy, PlantCARE, and NCBI. Detailed information about gene structure, phylogenetic trees, syntenic relationships, and promoter distribution is provided for each barley single and family gene. BGFD also provides statistical analysis, including exon numbers, chromosome locations, and variant distributions. These annotations provide a foundation for further gene isolation and functional characterization.
3. The transcript abundances of barley family genes were quantified using an exhaustive collection of 13 available RNA-seq datasets consisting of 413 samples with replicates. Expression profiles can be easily extracted to allow investigators to explore spatial-temporal and stress-induced expression profiles and biological functions of candidate genes. BGFD also features massive whole-exome resequencing (220 accessions) and pan-genome (22 accessions) datasets that could be used to evaluate both nucleotide and structural variants. The nucleotide and structural variations could be useful for the molecular breeding and characterization of functional genes with important agronomic traits in barley.
4. The BGFD interface is modern and accessible, allowing users to browse, search, and download areas of interest easily and effectively. The proposed platform enables data visualization in different forms. Our database also realizes practical functions such as keyword retrieval, BLAST alignment, and JBrowse browsing. The external links allow users to access other resources, thus adding or verifying gene family information to improve the accuracy of the data in BGFD.

## Conclusions

The increasing volume of multi-omics data provides a valuable source of information for studies of barley gene families. We constructed BGFD (http://barleygfdb.com) to facilitate the use of the comprehensive information mined from the continually growing amount of multi-omics data. We hope that BGFD will provide a valuable resource for future researchers and breeders interested in identifying candidate genes and functionally exploring important agronomical traits in barley. Given that the amount of omics data continues to grow at a rapid pace, we plan to continuously collect and share multi-omics information, especially epigenomic, proteomic, and metabolomic data, by incorporating this information into BGFD to ensure that our platform is as comprehensive and up-to-date as possible. In addition, more web-based practical tools for conducting studies of barley gene families will be developed and incorporated into BGFD in the future.

## Supplementary Information

**Additional file 1: Table S1.** Sample information and accession number of the RNA-seq data downloaded from the NCBI SRA database.

## Data Availability

Data pertaining to the study have been included in the article, and further inquiries can be directed to the corresponding authors. The barley genomes were downloaded from the given links: http://webblast.ipk-gatersleben.de/barley_ibsc/ (Morex V1), https://doi.org/10.5447/IPK/2019/19 (Morex V2), https://doi.org/10.5447/ipk/2021/3 (Morex V3), http://viewer.shigen.info/barley/index.php (OUH602), http://viewer.shigen.info/harunanijo/index.php (Haruna Nijo). The barley pan-genome project was downloaded from IPK database. https://barley-pangenome.ipk-gatersleben.de. The gene expression data and exome-capture resequencing data were downloaded from the NCBI database (http://www.ncbi.nlm.nih.gov/geo/) under BioProject accession number PRJEB14349, PRJEB13621, PRJEB18276, PRJNA382490, PRJNA496380, PRJNA428086, PRJEB12540, PRJNA324116, PRJNA400519, PRJNA704034, PRJNA439267, PRJNA744693, PRJNA728113 and PRJEB8044. Users can access our database through the following link: BGFD (http://barleygfdb.com).

## Abbreviations

ABA: Abscisic Acid
AP2/ERF: APETALA2/Ethylene-Responsive Factor
BAM: Binary Alignment Map
BGFD: Barley Gene Family Database
bHLH: Basic Helix-Loop-Helix
BR: Brassinosteroid
CDS: Coding Sequence
CNV: Copy Number Variant
FPKM: Fragments Per Kilobase Per Million Reads
GA: Gibberellin
GRAVY: Grand Average of Hydropathicity
GSDS: Gene Structure Display Sever
GTF: Gene Transfer Format
HiFi: High-Fidelity
HMM: Hidden Markov Model
HSF: Heat Shock Transcription Factor
II: Instability Index
Ka: Non-synonymous Substitution Rate
Ks: Synonymous Substitution Rate
MAF: Minor Allele Frequency
MEME: Multiple Expectation Maximization for Motif Elicitation
MW: Molecular Weight
NAC: NAM/ATAF/CUC
NCBI-CDD: National Center for Biotechnology Information–Conserved Domain Database
NJ: Neighbor-Joining
nsLTPs: Non-specific Lipid Transfer Proteins
OG: Ortholog Group
PAV: Presence/Absence Variation
pI: Theoretical Point
SNP: Single Nuclear Polymorphism
SPL: SQUAMOSA-Promoter Binding Like
SRA: Sequence Reading Archive
SSD: Small-Scale Duplication
TF: Transcription Factor
VCF: Variant Call Format
WGM: Whole-Genome Multiplication
XTH: Xyloglucan Endotransglucosylase/Hydrolase
